# Supplementary material for: Potential of methanol extracts of Nephelium lappaceum (Sapindaceae) and Hyphaene thebaica (Arecaceae) as adjuvants to enhance the efficacy of antibiotics against critical class priority bacteria
Source: PLoS One. 2025 Feb 12;20(2):e0314958. doi: 10.1371/journal.pone.0314958 (PMC11819497; doi:10.1371/journal.pone.0314958)
Supplement: S1 Table — (DOCX) [file pone.0314958.s001.docx]

**Supporting information**

**S1 Table.** **Common characteristics of critical-class priority bacteria used**

| **Bacteria species** | **Strain/isolates** | **Characteristics** |
| --- | --- | --- |
| *Staphylococcus aureus* | MRSA4 | Methicillin-resistant *Staphylococcus aureus* clinical isolate |
|  | MRSA6 | Methicillin-resistant *Staphylococcus aureus* clinical isolate |
| *Pseudomonas aeruginosa* | PA01 | *Pseudomonas aeruginosa* reference strain |
|  | PA124 | MDR clinical strains of *Pseudomonas aeruginosa* |
| *Escherichia coli* | AG100 | Wild type *E. coli*- k 12 |
|  | ATCC10536 | Reference strain of *E. coli* |
| *Klebsiella pneumoniae* | KP63 | Clinical MDR isolate, TET^R^ , CHL^R^ , AMP^R^ , and ATM^R^ of *Klebsiella pneumoniae* |
|  | ATCC11296 | Reference strain of *K. pneumoniae* |
| *Enterobacter aerogenes* | EA3 | Clinical strain *Enterobacter aerogenes* |
|  | EA27 | Clinical MDR isolate of *Enterobacter aerogenes* exhibiting energy-dependent norfloxacin and chloramphenicol efflux with KAN^R^ and AMP^R^ and NAL^R^ and STR^R^ and TET^R^ |

TET^R^, CHL^R^, AMP^R^, ATM^R^, KAN^R^; NAL^R^, and STR^R^ resistance to Tetracycline, Chloramphenicol, Ampicillin, Aztreonam, Kanamycin, Nalidixic acid, Streptomycin, respectively. MDR Multidrug resistant.
