# Supplementary material for: Potential of methanol extracts of Nephelium lappaceum (Sapindaceae) and Hyphaene thebaica (Arecaceae) as adjuvants to enhance the efficacy of antibiotics against critical class priority bacteria
Source: PLoS One. 2025 Feb 12;20(2):e0314958. doi: 10.1371/journal.pone.0314958 (PMC11819497; doi:10.1371/journal.pone.0314958)
Supplement: Attachment — Submitted filename: Review comment MF.docx [file pone.0314958.s002.docx]

Review Comment

The authors have examined the efficacy of methanolic extracts from two food plants (Nephelium lappaceum and Hyphaene thebaica), alone and in combination with antibiotics, against normal and resistant bacteria strains. Below are some comments

Result section :

- The authors do not indicate the MIC unit of the reference antibiotic (Imipenem). Is it also in (μg/mL)?
- It would be possible to use the most recent MIC classification scales, which reflect the current level of bacterial virulence in clinical practice.
- The figures shown are not numbered.
- The first figure is not numbered on the x-axis.
