# Supplementary material for: Potential of methanol extracts of Nephelium lappaceum (Sapindaceae) and Hyphaene thebaica (Arecaceae) as adjuvants to enhance the efficacy of antibiotics against critical class priority bacteria
Source: PLoS One. 2025 Feb 12;20(2):e0314958. doi: 10.1371/journal.pone.0314958 (PMC11819497; doi:10.1371/journal.pone.0314958)
Supplement: Attachment — Submitted filename: Response to Reviewers.docx [file pone.0314958.s003.docx]

Manuscript Number: PONE-D-24-16310

**Dear Editor,**

**Dear Reviewers,**

We are grateful for the constructive and instructive comments made to improve the quality of our manuscript. We are very well aware that this review has been a significant time investment and therefore especially appreciate the editor and reviewers' feedback and commitment. We have provided below point-by-point responses to the editor and reviewers’ comments.

During the revision process, we addressed all comments, and the vast majority of them led to additions or clarifications in the text. Additional references have been included. On the other hand, two tables were moved from the supplementary material to the main manuscript text, as suggested by reviewer#1, and then the tables in the main text have been renumbered accordingly.

Furthermore, the most recent interpretive criteria of the antibacterial activity of plant extracts were inserted and used, as suggested by reviewer#2, which also responded to the comments made by reviewer#4 about statistical or data analysis. Many parts of the text, particularly the discussion, have been clarified following related remarks and pertinent questions of reviewer#5 and details have been added where necessary. We are currently unable to add further testing such as antioxidant actions suggested by reviewer#4 or GC-MS analysis suggested by reviewers #4 and #5. Detailed responses were provided to justify this. However, in response to the need for GC-MS analysis, we have added details of key phytoconstituents previously reported in the studied plants, with most results corroborating our qualitative phytochemical analysis. We expect that this will be sufficient at this point.

With the modifications performed following the very helpful remarks and questions of the reviewers, we have now an improved manuscript. Finally, the manuscript has been carefully formatted following the journal requirements, as requested by the editor.

**All modified sections or statements are written in red and highlighted in yellow in the track-change version of the manuscript.**

We hope that these substantial revisions are satisfactory for the reviewers and the editor.

Best regards,

**Armel J. Seukep**, PhD, on behalf of the author team.

**REPLY TO JOURNAL REQUIREMENTS:**

When submitting your revision, we need you to address these additional requirements.

1. Please ensure that your manuscript meets PLOS ONE's style requirements, including those for file naming. The PLOS ONE style templates can be found at

https://journals.plos.org/plosone/s/file?id=wjVg/PLOSOne_formatting_sample_main_body.pdf and

<https://journals.plos.org/plosone/s/file?id=ba62/PLOSOne_formatting_sample_title_authors_affiliations.pdf>

**Response**: Dear Editor, the manuscript was revised to meet PLOS ONE's style requirements as displayed in the PLOS ONE style templates.

2. In your Methods section, please provide additional information regarding the permits you obtained for the work. Please ensure you have included the full name of the authority that approved the field site access and, if no permits were required, a brief statement explaining why.

**Response**: Dear Editor, no permits were required for the work. Investigations were performed within the Laboratory of Biochemistry of the Faculty of Health Sciences of the University of Buea (Cameroon) led by myself, and the Laboratory of Cancer Research of the University of Dschang (Cameroon) under the chair of Prof. Kuete Victor, who is our research group leader and co-author of this submission.

However, since the studied plants were identified on field sites, out of our leadership, the names of experts (botanists) who identified/authenticated the plant material were provided in the manuscript's ‘Plant material and extraction’ section. The plant samples are commonly edible plants, not listed as endangered species, and were collected in fresh form in the markets, requiring no special permit.

3. We note that your Data Availability Statement is currently as follows: [All relevant data are within the manuscript and its Supporting Information files.]

Please confirm at this time whether or not your submission contains all raw data required to replicate the results of your study. Authors must share the “minimal data set” for their submission. PLOS defines the minimal data set to consist of the data required to replicate all study findings reported in the article, as well as related metadata and methods (https://journals.plos.org/plosone/s/data-availability#loc-minimal-data-set-definition).

For example, authors should submit the following data:

- The values behind the means, standard deviations and other measures reported;

- The values used to build graphs;

- The points extracted from images for analysis.

Authors do not need to submit their entire data set if only a portion of the data was used in the reported study.

If your submission does not contain these data, please either upload them as Supporting Information files or deposit them to a stable, public repository and provide us with the relevant URLs, DOIs, or accession numbers. For a list of recommended repositories, please see https://journals.plos.org/plosone/s/recommended-repositories.

If there are ethical or legal restrictions on sharing a de-identified data set, please explain them in detail (e.g., data contain potentially sensitive information, data are owned by a third-party organization, etc.) and who has imposed them (e.g., an ethics committee). Please also provide contact information for a data access committee, ethics committee, or other institutional body to which data requests may be sent. If data are owned by a third party, please indicate how others may request data access.

**Response**: Dear Editor, we confirm that our submission contains all raw data required to replicate the results of the study. Moreover, there are no ethical or legal restrictions on sharing data. The Data Availability Statement has been added to the manuscript, before References, as follows:

‘We confirm that our submission contains all raw data required to replicate the results of the study. Moreover, there are no ethical or legal restrictions on sharing data.’

4. Please include captions for your Supporting Information files at the end of your manuscript, and update any in-text citations to match accordingly. Please see our Supporting Information guidelines for more information: <http://journals.plos.org/plosone/s/supporting-information>.

**Response**: Dear Editor, the captions for the Supporting Information files have been added at the end of the manuscript. In-text citations and the reference list were updated based on the additional information in the manuscript.

**REPLY TO THE REVIEWERS’ COMMENTS:**

**Reviewer #1:**

Methodology:
"All bacterial isolates were cultured on Mueller Hinton agar (MHA) (Liofilchem S.r.l., Italy) and the microdilution testing was done using Mueller Hinton broth (MHB) (Titan Biotech Ltd., India) to determine the test samples' minimum inhibitory concentration (MIC), minimum bactericidal concentration (MBC), and activity increase factor (AIF) (following combination assays)."
Q: The combination assay should be elaborated in the methodology section.

**Response**: Dear Reviewer, thanks for the observation. The full description of the protocol for combination assay is provided in the ‘Plant extract/antibiotic combination’ section of the methodology.

Results:
Table 1- Why MBC values are not stated for all the tested microorganisms? Hence it has resulted in not having the MBC/MIC ratio values for respective bacterial isolates.

**Response**: Dear Reviewer, thanks for the pertinent remark. Plant extracts were tested at 2048 µg/mL as the highest concentration. Therefore, MBC values were not stated for all extracts having a MBC > 2048 µg/mL. Hence, since exact MBC values were not recorded, this prevented us from calculating the MBC/MIC ratio. For clarification, the symbol (-) was added to Table 1 (besides MIC values) and the footnotes to indicate MBC values > 2048 µg/mL.

Why only one Antibiotic (Imipenem) has been used as a reference antibiotic for all the tested microorganisms? Explain this.

**Response**: Dear Reviewer, thanks for the question. Imipenem has been chosen as the reference antibiotic for all the tested microorganisms because of its wide spectrum of antibacterial activity against both gram-negative and gram-positive aerobic bacteria, including many multi-drug resistant strains, as used in our study.

This justification and corresponding citation were added in the MIC determination section of the methodology.

Combination Testing results should be provided as part of the results and not as Supplemental information 2 and 3.

**Response**: Dear Reviewer, thanks for the constructive remark. The corresponding results from supplemental information 2 and 3 were moved to the main text of the results section. They have been renamed as Table 2 and Table 3.

**Reviewer #2:**

The authors have examined the efficacy of methanolic extracts from two food plants (*Nephelium lappaceum* and *Hyphaene thebaica*), alone and in combination with antibiotics, against normal and resistant bacteria strains. Below are some comments
Result section :
• The authors do not indicate the MIC unit of the reference antibiotic (Imipenem). Is it also in (μg/mL)?

**Response**: Dear Reviewer, thanks for the remark. The MIC of the reference drug Imipenem is in μg/mL, and this detail has been added to Table 1.

• It would be possible to use the most recent MIC classification scales, which reflect the current level of bacterial virulence in clinical practice.

**Response**: Dear Reviewer, thanks for the constructive comments. We updated the MIC classification of the antibacterial activity of botanicals to the most recent ones. Detailed information was provided in the new section ‘Data interpretation/analysis’ of the methodology, then, consistently used in the discussion section.

• The figures shown are not numbered.

**Response**: Dear Reviewer, thanks for the observation. The figures have been double-checked and numbered consistently.

• The first figure is not numbered on the x-axis.

**Response**: Dear Reviewer, thanks for the remark. The x-axis is numbered, however, there is a difference in the x-axis interval of the various figures to facilitate the appearance and reading of resulting interactions.

**Reviewer #3:**

This work reports the Potential of methanol extracts of *Nephelium lappaceum* (Sapindaceae) and *Hyphaene thebaica* (Arecaceae) as adjuvants to enhance the efficacy of antibiotics against critical class priority bacteria.

The work is meaningful and interesting.

**Response**: Dear Reviewer, we are grateful for your appreciation of our work. Thanks.

In the "Introduction" or "Experimental Section", the authors need to provide or add a more detailed explanation about exploring the antibacterial activity of plants which in this study was used to methanol extracts. Here are some papers that can be used by the authors to find out more about the antibacterial activity of plants and need to be added and cited by the authors in this manuscript:

doi: 10.1016/j.sjbs.2021.10.057
doi: 10.1155/2021/6663399
doi: 10.1038/s41598-023-32900-1

**Response**: Dear Reviewer, thanks for the suggested references. More details were added to justify the antibacterial activity of plants in the introduction, with additional references amongst which the three references suggested.

**Reviewer #4:**

The current Manuscript Number: PONE-D-24-16310; entitled: Manuscript Title: Potential of methanol extracts of *Nephelium lappaceum* (Sapindaceae) and *Hyphaene thebaica* (Arecaceae) as adjuvants to enhance the efficacy of antibiotics against critical class priority bacteria

The current manuscript contains a good idea and I have the following comments:
- Please add statistical analysis in the methods section and explain the significance along the whole manuscript.

**Response**: Dear Reviewer, thanks for valuing our research and the pertinent remark. The ‘data interpretation/analysis’ section was added to the methodology section, then the significance was consistently used to analyze and discuss the results obtained.

- It is essential to identify the bioactive compounds of extracts using one of the chromatographic analyses e.g.: Gas chromatography and link the antimicrobial activity to these compounds.

**Response**: Dear Reviewer, thanks for the insightful comment. We agree with the suggestion, but due to limited equipment, we were unable to perform further phytochemical analyses such as GC-MS. However, we carried out a preliminary screening of the test samples which provided an overview of the major classes of bioactive secondary metabolites from the plants that may account for the recorded activities. The in-depth analysis of the bioactive extracts is in perspective and we expect to develop future collaboration to achieve it.

However, in addition to the preliminary phytochemical screening we performed, we reviewed the major phytochemicals previously identified from the studied plants and highlighted the link to their antibacterial effects. We expect that this can be sufficient at this level.

- I highly recommend to examine antioxidant actions of extracts and compare all of them.

**Response**: Dear Reviewer, thanks for the pertinent recommendation, we appreciate it. Although the antioxidant actions would be an important addition to the current work, we currently focus in our group on the antibacterial actions of herbals against multi-drug resistant phenotypes. However, we appreciate the suggestion and may consider it as a perspective for further biopharmaceutical examination of the plants of interest.

- Please rewrite the manuscript sections after adding the suggested experiments and statistics.

**Response**: Dear Reviewer, thanks for the comments. The manuscript was revised and details about data interpretation/analysis (statistics) were added.

**Reviewer #5:**

Dear Reviewer, we especially thank you for the thorough and constructive questions. These permit us to significantly improve the discussion section of the manuscript, where most of the answers (when not yet previously explained) provided here have been added.

1. Why do various portions of *Nephelium lappaceum* and *Hyphaene thebaica* have distinct antibacterial effects against multidrug-resistant bacteria?

**Response**: Dear Reviewer, thanks for the pertinent question. The differences recorded in the antibacterial effects of various parts of the test plants can be explained by the differences in phytochemicals content, both qualitatively and quantitatively, the possible interactions amongst the phytoconstituents, and the antibacterial modes of action. This explanation has been added to the discussion section.

2. What caused *Nephelium lappaceum* leaf extract to have the greatest antibacterial activity against all multidrug-resistant strains? What are its mechanisms?

**Response**: Dear Reviewer, thanks for the insightful question. *Nephelium lappaceum* leaf extract may contain key constituents capable of neutralizing drug-resistant components in studied bacteria. In the present study, the phytochemical analysis of the extract from *Nephelium lappaceum* leaves revealed the presence of steroids, phenolics, tannins, and saponin, which are classes of secondary metabolites from which well-established plant antibacterial drugs have been reported against multi-drug resistant bacteria strains.

In addition, possible mechanisms of its antibacterial actions were unveiled in our research. Indeed, the findings revealed the potential of the extract to alter the bacterial cell membrane and induce leakage of genetic material, which could destroy the bacteria cell and kill the bacteria. Furthermore, the extract has been revealed to inhibit the H^+^-ATPase bacterial pump, limiting the provision of energy for cellular function and proliferation of bacteria.

3. What causes the extracts' MIC and MBC values to vary for various bacterial strains?

**Response**: Dear Reviewer, thanks for the question. The difference may be probably due to each pathogen's intrinsic characteristic and specific resistance feature. Moreover, the various phyto-components in each extract may have different antibacterial targets. This explanation has been added to the discussion section.

4. Given the synergy between plant extracts and antibiotics, how can *Nephelium lappaceum* and *Hyphaene thebaica* extracts boost antibiotic efficacy?

**Response**: Dear Reviewer, thanks for the pertinent question. The bacteria used in the study are known to over-express active efflux which is one of the main resistance strategies resulting in multiple-drug resistance in bacteria. The noteworthy synergistic interactions recorded may suggest that the extracts block the efflux machinery in bacteria thereby favoring an increased concentration of antibiotics within the bacterial cell, enough to produce inhibitory or killing effects. Moreover, extracts may alter the bacterial cell membrane, thereby increasing the cell permeability to various agents including antibiotics. Furthermore, possible interactions with key bacterial enzymes (such as beta-lactamases) can inhibit their activities thereby preventing further destruction of antibiotics having beta-lactam moiety such as imipenem used in our study. This additional explanation has been added to the discussion section.

5. What does the MBC/MIC ratio of < 4 indicate for most extracts, and how does it affect their bactericidal vs bacteriostatic properties?

**Response**: Dear Reviewer, thanks for the question. The extracts having MBC/MIC ratio ≤ 4 were considered to have a bactericidal effect. This suggests the ability of the specified extracts to produce drug concentrations that will kill 99.9% of the organisms exposed. If the MBC/MIC ratio is high (MBC/MIC >4), it may be impossible to safely deliver quantities of the antibiotic that kill 99.9% of the bacteria, and the drug is classified as bacteriostatic. This explanation has been added to the discussion section.

6. How does *Nephelium lappaceum* leaf extract's capacity to lower pH by inhibiting H⁺-ATPase relate to its antibacterial effectiveness?

**Response**: Dear Reviewer, thanks for the question. The proton pumps (H⁺-ATPase) in bacteria are responsible for maintaining the homeostatic media required to function correctly. The inhibition of H^+^-ATPase-dependent proton pumps can lead to a decrease in the survival of bacteria, as they require a certain level of energy in the medium for metabolism, growth, and multiplication. This explanation was already stated in the discussion section.

7. How can certain secondary metabolites (saponins, phenols, tannins, steroids, alkaloids) affect the antibacterial activity of plant extracts, and how could their presence or absence affect results?

**Response**: Dear Reviewer, thanks for the question. Phytochemicals of the major secondary metabolites group identified including saponins, phenols, tannins, steroids, and alkaloids have proven to possess potent antibacterial activity. The absence of one or more of these secondary metabolites can dictate how efficiently the extract could act against bacteria. However, the quality, quantity, and possible interactions amongst compounds in the phytocomplexes are also key parameters to consider.

8. What does the absence of antagonistic interactions between plant extracts and antibiotics mean for future combination treatment methods for multidrug-resistant bacterial infections?

**Response**: Dear Reviewer, thanks for the insightful question. Antagonism between two agents may aid in devising treatment strategies specifically aimed at delaying the emergence of resistance. However, the absence of antagonistic interactions between plant extracts and antibiotics is a promising result, which suggests that the studied extracts either maintain the efficacy of antibiotics (indifference or no interaction) or improve it (synergistic interaction), as obtained in our study. This additional explanation was added in the discussion section.

9. How do *Nephelium lappaceum* and *Hyphaene thebaica* phytochemical profiles and extraction yields differ, and how could they affect their antibacterial applications?

**Response**: Dear Reviewer, thanks for the insightful question. The initial powder’s weight differs from one extract to another (which depends on the initial amount of fresh plant material collected), which can partially explain the differences in extraction yield obtained. In addition, the differences in both extraction yield and phytochemical profiles can be explained by the nature of the solvent used, the differences in plant parts, and the intrinsic phytochemical constitution of each plant or plant part. Indeed, some plant parts, such as seeds and peels may have concentrated amounts of phytochemical agents or contain oils (oily extracts usually have higher weight, which therefore influence the extraction yield). Moreover, the extraction yield is generally related to the amount of bioactive ingredients or secondary metabolites extracted from the plant. However, there is no direct correlation between higher or lower extraction yield and the antibacterial properties of an herbal. Indeed, the type of phytochemical in a plant extract and the possible interactions amongst the phytoconstituents are additional key parameters that could significantly influence the antibacterial efficacy of the plant extract. For instance, in our study, the extract (leaves of *Nephelium lappaceum*) with the least extraction yield produced the best antibacterial efficacy. These detailed explanations are provided in the discussion section.

10. What further study is required to understand the cellular and molecular mechanisms of *Nephelium lappaceum* and *Hyphaene thebaica* extracts' antibacterial properties, especially their impact on bacterial cell membranes and metabolic processes?

**Response**: Dear Reviewer, thanks for the pertinent question. Our study provides a sound experimental baseline to consider *Nephelium lappaceum* and *Hyphaene thebaica* extracts for further investigations, which could include cellular and molecular mechanisms of the crude extract and further identified/isolated phytochemicals targeting key components of the bacterial cell such as membrane and cytoplasmic proteins, cell wall constituents (peptidoglycan), DNA, extracellular and intracellular bacterial enzymes, ribosomal function, and folate synthesis. These are currently some perspectives for our future work as stated in the discussion section.

11. The author needs to provide GC-MS data of the active molecules present in the extracts.

**Response**: Dear Reviewer, thanks for the pertinent comment. We agree with the suggestion, but due to limited equipment, we were unable to perform further phytochemicals such as GC-MS. However, we performed a preliminary screening of the most active sample which provided an overview of the major classes of bioactive secondary metabolites from the plants that may account for the recorded activities. The in-depth analysis of the bioactive extracts is in perspective and we expect to develop future collaboration to achieve it. However, in addition to the preliminary phytochemical screening carried out in our research, we reviewed the major phytochemicals previously identified from the studied plants and highlighted the link to their antibacterial effects. We expect that this can be sufficient at this level.

12. It is essential to compare the biological activity of the extract with that of the pure compound identified through GC-MS analysis. This comparison will add novelty to the study; otherwise, using extracts alone lacks significance, as there are already numerous studies (more than 1000 publications) focused on extract-based biological activities.

**Response**: Dear Reviewer, thanks for the constructive comment. As stated in the previous comment, we agree with the suggestion, but due to limited equipment, we were unable to perform further phytochemicals such as GC-MS. However, we carried out a preliminary screening of the most active sample which provided an overview of the major classes of bioactive secondary metabolites from the plants that may account for the recorded activities. In addition, in the discussion section, we reviewed the major phytochemicals identified previously from the studied plants and their documented antibacterial effects. We expect that this can be sufficient at this level of investigation.
